# Double-Butter: A Cementation Technique That Significantly Reduces Lipid Contamination of the Tibial Baseplate in Total Knee Arthroplasty

**DOI:** 10.1016/j.artd.2024.101574

**Published:** 2024-12-07

**Authors:** Jacob M. Wilson, Aleksander P. Mika, William W. Gilbert, J. Bohannon Mason, Gregory G. Polkowski, J. Ryan Martin

**Affiliations:** a Department of Orthopedic Surgery, Emory University, Atlanta, GA, USA; b Department of Orthopedic Surgery, Vanderbilt University, Nashville, TN, USA; c OrthoCarolina Hip and Knee Center, Charlotte, NC, USA

**Keywords:** Aseptic loosening, Lipid contamination, Cement technique, Double butter

## Abstract

**Background:**

Aseptic loosening is the most common aseptic failure modality following total knee arthroplasty. Recent literature suggests that the implant-cement interface is the “weak-link” in fixation and lipid contamination may drive this debonding pattern. Therefore, the purpose of this study was to determine if the “double-butter” technique would significantly decrease lipid contamination of the tibial tray.

**Methods:**

Transparent acrylic models of 7 different tibial baseplates were created to allow for direct visualization of fluid contamination of the implant-cement interface during experimental cementation. Three cementation techniques were then employed in triplicate for each implant: coating only the tibia (“single butter”) and coating of the tibia and baseplate (with and without keel included; “double-butter”). A dye was added centrally to simulate lipid contamination. After each trial, the degree of implant-cement contamination was calculated. Standard statistical analyses were conducted.

**Results:**

With the double-butter technique, there was a significant reduction in contamination for all studied implant designs (range: 0%-7%; *P* < .05) and contamination was eliminated when the entire implant was coated prior to implantation. The single-butter technique resulted in contamination of 16%-43% of the tibial undersurface. There were significant differences in percent contamination between component designs (*P* < .05).

**Conclusions:**

Cementation technique and implant design each influenced baseplate lipid contamination. While significant differences were noted between keel geometries, we found that the double-butter technique effectively eliminated baseplate contamination, even in the most susceptible designs in this study. We therefore advocate for the incorporation of the double-butter technique to limit lipid contamination and potentially reduce aseptic tibial loosening.

## Introduction

Primary total knee arthroplasty (TKA) is well established as an effective operation. [1] While the lifetime risk of revision is low for the majority of patients, the prevalence of revision TKA continues to increase. [2,3] Despite advances in implant design and bearing surfaces, [4,5] aseptic loosening remains the leading indication for revision TKA. [6] Historically, loosening was associated with wear of conventional polyethylene and subsequent osteolysis. [7] However, with contemporary crosslinked polyethylene, this failure mode has decreased drastically. [5]

Mechanical debonding at the cement-component interface, however, has been an increasingly commonly reported entity in contemporary TKA. [[8], [9], [10]] Prior research found that in cases of aseptic loosening 94% occurred at the implant-cement interface, [11] even in cases of varus collapse. [12] For these reasons, both implant design and cementation technique have seen renewed focus to minimize these failure mechanisms. [11,13,14] Bili et al have shown that early application of polymethylmethacrylate to the metal substrate improves cement binding strength and that this adhesion strength can be deteriorated by in vitro lipid contamination. [15] Furthermore, it has been demonstrated that coating the back of the tibial component as well as the bony surface with cement (double-butter [DB]) optimizes cement penetration [14] and improves implant pull-out strength. [13] Martin et al have hypothesized that this may be related to cement-implant lipid contamination. [13,16]

Despite these prior findings, there is currently no gold standard cementation technique. [17,18] To this point, a recent survey of the American Association of Hip and Knee Surgeons membership revealed that there is high variability in surgeon cementation technique. [18] In particular, only 46% and 48% of surgeons reported coating the entirety of the bone and implant with cement on the tibia and femoral sides, respectively. [18] The purpose of the present study was to assess the impact of cement technique and implant keel design on lipid contamination of the underside of the tibial baseplate. We hypothesized that different implants would have variable susceptibility to lipid contamination and that this would be limited by a DB cementation technique.

## Material and methods

**Figure 1:**
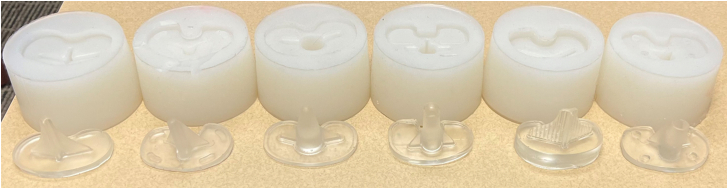
Acrylic tibial baseplates and molds created for experimental tibial implantation. Of note, pictured implants are photographed in no particular order and not all implants are pictured.

Institutional review board approval was not required for this study. Seven contemporary tibial baseplates were obtained and utilized to manufacture transparent, acrylic models of each tibial component (Fig. 1). These acrylic models were created for utilization in this study. The tibial component designs included: Zimmer-Biomet Persona (standard cemented tray and cemented keel tray, Zimmer-Biomet, Warsaw, IN), Zimmer-Biomet Vanguard (Zimmer-Biomet, Warsaw, IN), Depuy PFC Sigma fixed bearing (Depuy, Warsaw, IN), Depuy Attune (original and S+ tibial trays, Depuy, Warsaw, IN), and Stryker Triathlon (primary baseplate, Stryker, Mahwah, NJ). The tibial baseplate sizes chosen were of similar size. We also manufactured rubber molds to simulate a prepared bony surface for simulated implantation and cementation.

**Figure 2:**
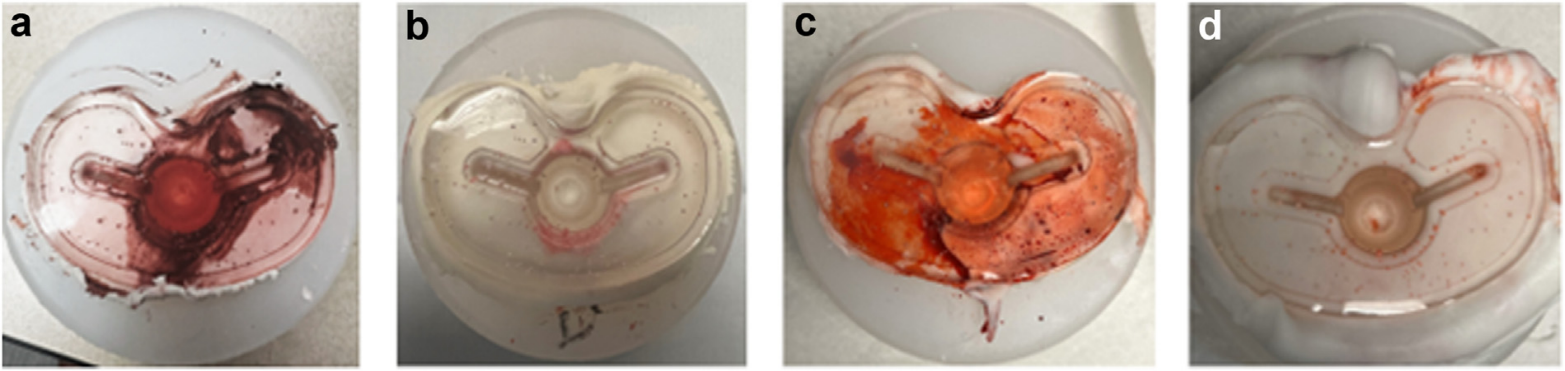
Comparison of results using the modeling dough (a, single butter; b, double-butter) and PMMA (SimplexP; C, single butter; D, double-butter). Note that when using real cement, the difference in fluid contamination was more dramatic than when using the modeling dough. PMMA, polymethylmethacrylate.

We chose to utilize a white modeling dough to replace cement. This dough has a consistency and appearance that is very similar to polymethylmethacrylate when it is in its working form. This methodological decision was made intentionally to eliminate any inconsistencies with regards to the viscosity of the cement at the time of simulated implantation between trials. In order to confirm that this methodological decision was appropriate, a trial with polymethylmethacrylate (SimplexP, Stryker), was conducted with a single-butter (SB) and DB trial. With real cement, the differences found between these 2 cementation techniques was more dramatic than that observed with the modeling dough (Fig. 2).

**Figure 3:**
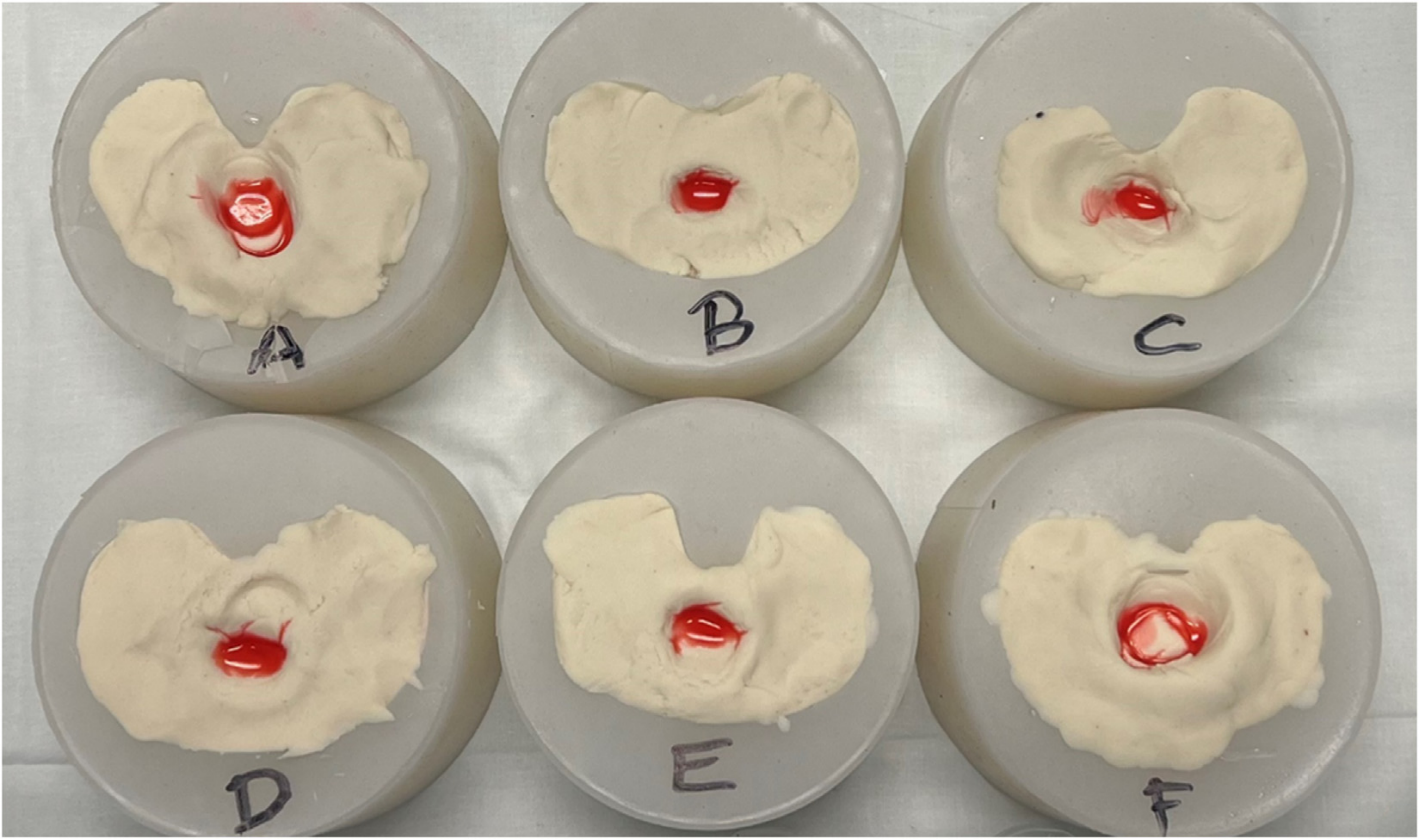
Photo of prepared cemented surface for experimental tibial implantation. This was created for each cementation technique trial. Cement was added to the backside of the implant as described for the 2 double-butter cohorts. Of note, only 6 of 7 tibial molds are shown.

Each implant was put through simulated implantation using 3 different cementation techniques. In the first, cement was applied to only the “bony” surface and not applied to the backside of the implant (SB). The second iteration consisted of coating the prepared “bony” surface and the underside of the tibial baseplate (but not the entire keel; DB without keel). The third iteration consisted of coating the entire implant (including the keel; DB with keel) and the entire “bony” surface. In each trial, prior to implantation, 3 drops (0.15 mL) of red dye (Food, Drug, and Cosmetic Reds 40 and 3; McCormick and Company, Hunt Valley, MD) were added to the top of the cement in the region of the keel to simulate lipid or fluid contamination that occurs routinely intraoperatively (Fig. 3). Each implant underwent simulated implantation with each of the above techniques in triplicate. After each simulated implantation, a photo was taken from directly above the implant.

Photos of each trial were then evaluated digitally utilizing ImageJ image processing software (version 1.54e; National Institutes of Health, Bethesda, USA). Contamination was measured on a percentage-based system, such that the contaminated area (ie, area where the dye was visualized) could be calculated as a percentage of the entire tibial baseplate. Given variable keel geometries and sizes, the area of the keel was subtracted from the area of the entire baseplate prior to calculation of this percent contamination. A photo of each trial was measured by 2 authors (AM and WG) and these measurements were averaged for data analysis.

### Statistical analysis

Interobserver reliabilities were calculated using the intraclass correlation coefficient and demonstrated near perfect agreement between author measurements (intraclass correlation coefficient = 0.98; 95% confidence interval 0.97-0.99). Descriptive statistics were utilized to quantify percent baseplate contamination by cementation technique and component type. One-way analysis of variance (ANOVA) tests were conducted to detect differences in percent contamination by implant design. Post-ANOVA Tukey analysis was utilized to compare different implant designs. This analysis was adjusted for multiple comparisons. Paired student’s *t*-tests were utilized to compare differences between cementation techniques. A *P* value of < .05 was considered statistically significant. Statistical analysis was performed using R, version 4.1.2 (R Core Team, R Core Team, R Foundation for Statistical Computing, Vienna, Austria).

## Results

### SB cementation technique

**Figure 4:**
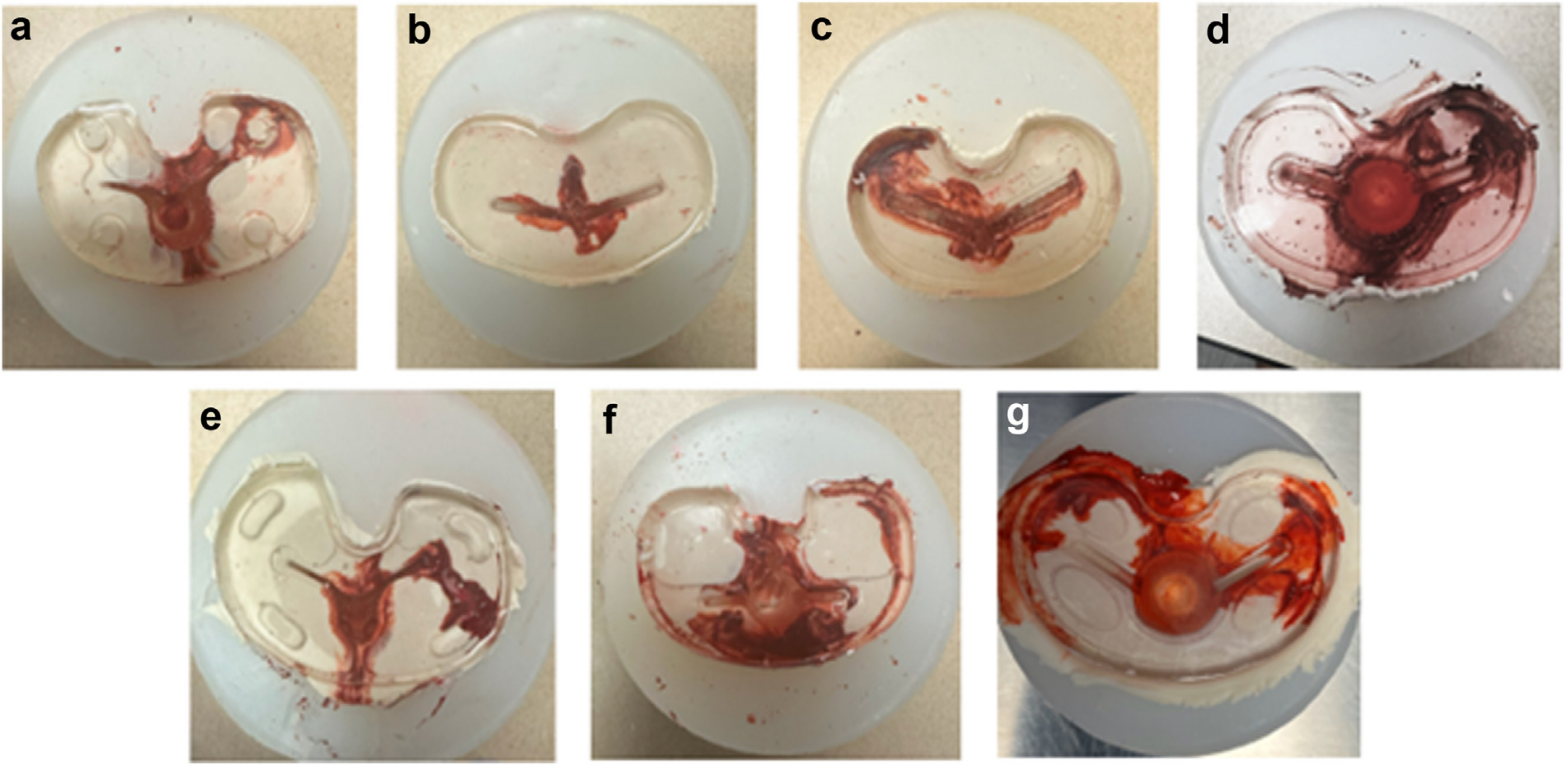
Photos demonstrating fluid contamination at the cement-implant interface during single-butter cementation technique.

The mean percentage of baseplate lipid contamination when only the tibial plateau was covered with cement was 22%, 16%, 37%, 43%, 37%, 35%, and 30% for implants A, B, C, D, E, F, and G, respectively (Table 1, Fig. 4). There was a statistically significant difference among percent contamination among implants (*P* = .04). Post-ANOVA Tukey analysis found a significant difference between implant D and implant B (*P* = .04). There were no other pairwise comparisons that were individually significant.

**Table 1:** Average fluid contamination of tibial baseplate based on cementation technique.

	Cementation technique			
	Single-butter	Double-butter (without keel)	Double-butter (with keel)	*P* value^a^
Implant A	22.19%	0.06%	0%	.009
Implant B	15.53%	1.03%	0%	.009
Implant C	36.68%	0.12%	0%	.03
Implant D	42.82%	7.45%	0%	.02
Implant E	36.71%	0.74%	0%	.03
Implant F	34.72%	2.51%	0%	.04
Implant G	30.36%	0.00%	0%	.01

a *P*-value <.05 was considered significant.

### DB cementation technique (baseplate only, keel not included)

**Figure 5:**
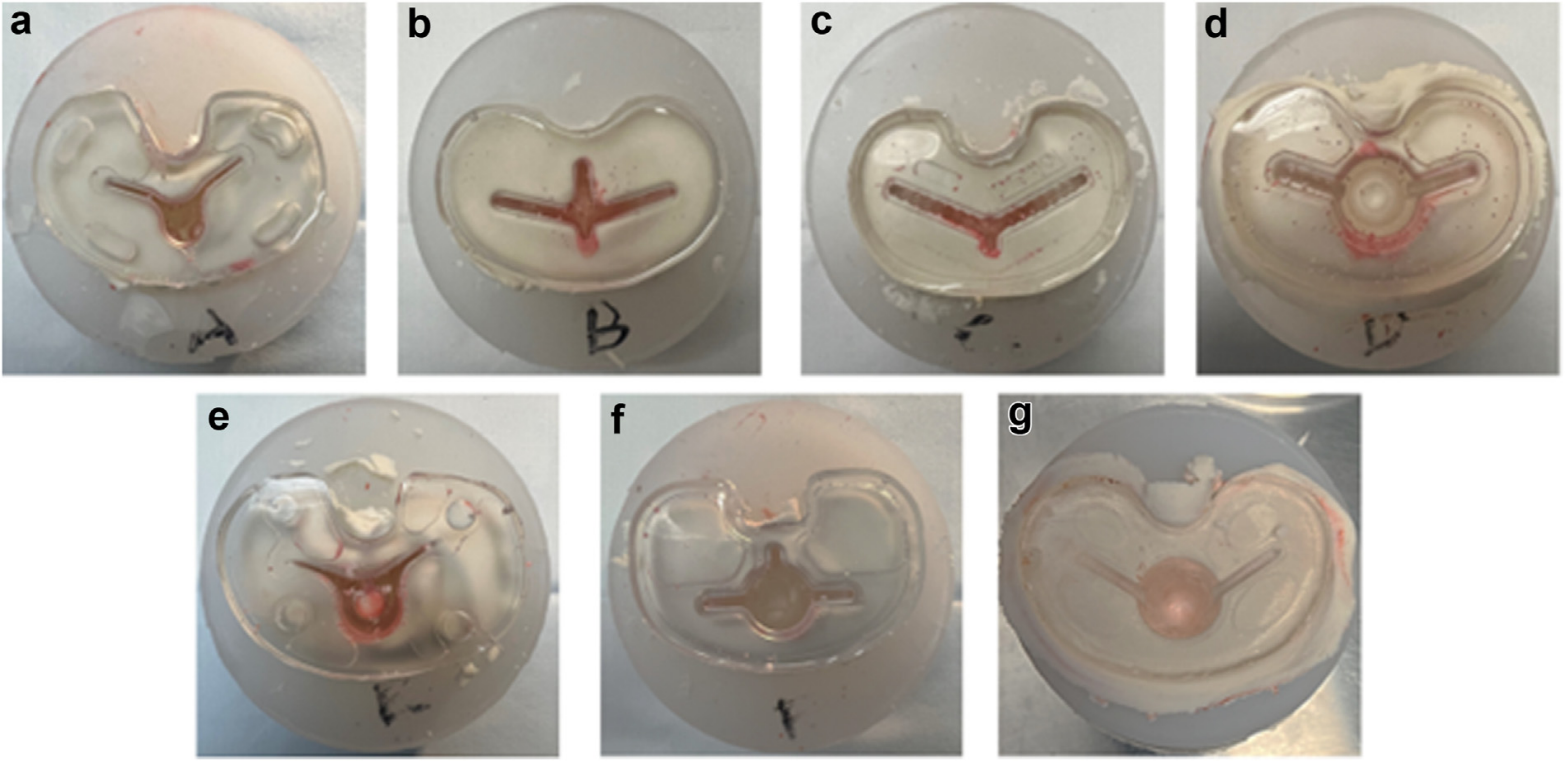
Photos demonstrating reduced fluid contamination when a “double-butter” technique is utilized. When the entire implant (baseplate and keel) was coated, there was no observed fluid contamination.

The mean baseplate contamination when both the underside of the baseplate and tibial plateau were coated with cement was 0.06%, 1%, 0.1%, 7.5%, 0.7%, 2.5%, 0.1%, and 0%, for implants A, B, C, D, E, F, and G, respectively (Table 1, Fig. 5). There was a significant difference among tested implants (*P* < .001). Tukey post-ANOVA analysis demonstrated that there were pairwise differences between implant D and every other included implant (*P* ≤ .01 for all). No other pairwise differences were noted.

### DB cementation technique (baseplate and keel coated)

When the entire underside of the implant was coated with cement, there was no observed fluid contamination of the implant-cement interface noted in any of the included baseplates with any trials. Put another way, fluid contamination at the implant-cement interface was eliminated for all designs and there was, therefore, no difference in percent contamination among designs with this technique.

### SB vs DB

**Figure 6:**
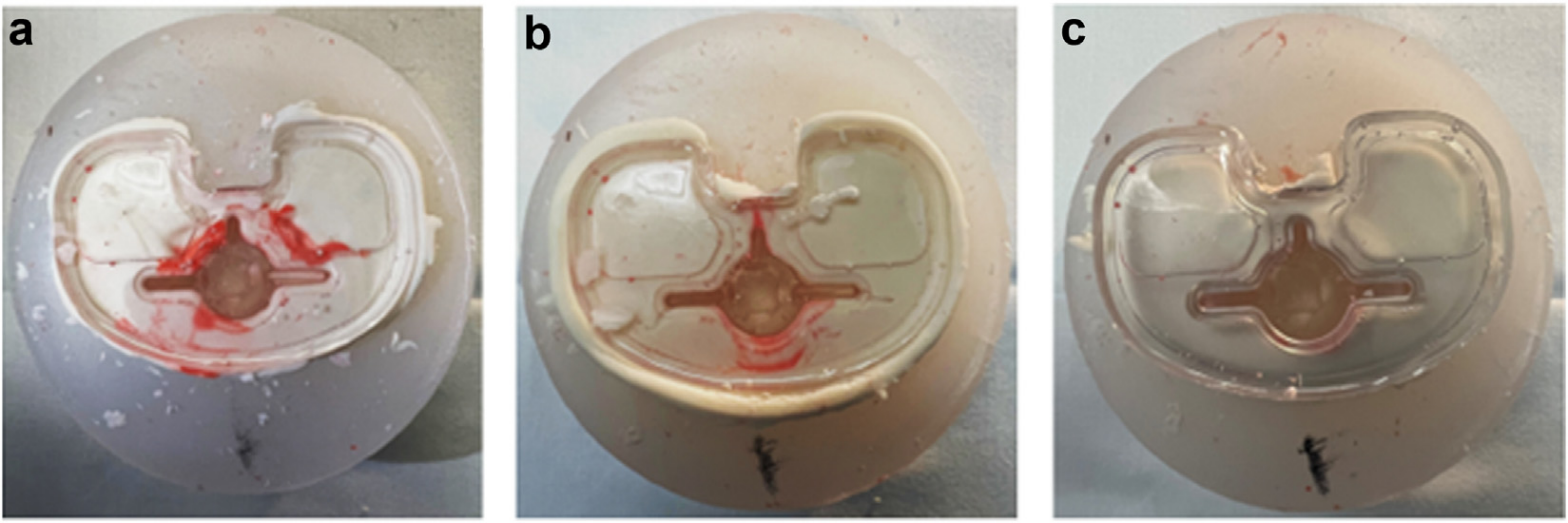
Example of fluid contamination with various cementation techniques: (a) single-butter, (b) double-butter without keel included, (c) double-butter with keel included. Note the difference in fluid contamination between a and b, with complete fluid contamination elimination when the entire implant was coated.

Paired student’s *t*-tests demonstrated that there was a significant reduction in baseplate contamination with a DB technique when compared to SB technique in all studied designs. This was significant for all studied designs (*P* ≤ .04 for all comparisons; Table 1, Fig. 6).

## Discussion

While polyethylene wear and osteolysis are less frequently encountered with highly cross-linked polyethylene, [4,5] aseptic loosening remains a contemporary issue. [6] A distinct form of aseptic loosening, tibial implant debonding, has been described with modern implants. [[8], [9], [10]] Additionally, prior work on aseptic loosening has demonstrated that the vast majority of the time, loosening occurs at the cement-implant interface. [11] It has been hypothesized that this may be related to implant-cement interface lipid contamination. [13] Cementation technique may play an important role in limiting lipid contamination but currently, there is no gold-standard cementation technique and there is, consequently, significant surgeon variation. [17,18]

In the present study, we utilized transparent acrylic tibial component models to examine the influence of cementation technique on fluid contamination of the implant-cement interface. The results of this study indicate that even with only minimal fluid present at the cement interface, use of a bone-only cementation technique results in contamination of a large portion of the cement-implant interface with tibial implantation. However, simply applying cement to underside of the tibial component eliminated contamination of the cement-implant interface and inter-component variations. It should be noted that this result was optimized with applying cement to the entire undersurface of the component (including the keel), rather than only the baseplate. Given the variance in contamination among differing implant designs, it does appear that keel geometry and component design play a role in contamination of the cement-implant interface with some designs being more susceptible than others. These findings warrant further discussion.

One of the most important findings of this study was that despite only adding 3 drops of fluid to the cement interface—a volume of contamination consistent with clinical in-vivo occurrences—a substantial portion of the tibial surface area was contaminated when only the bone was coated with cement. The importance of this finding is underscored by the 2022 survey of American Association of Hip and Knee Surgeons membership which found that nearly 50% of surgeon respondents utilize this technique. [18] Previous work has demonstrated that use of a DB cementation technique increases implant fixation strength and has theorized that this may be due to less lipid contamination. [14] In this study, coating the backside of the implant and the bone with cement (DB technique), eliminated this fluid contamination at the implant cement interface, suggesting that a simple practice modification may reduce the risk of implant-cement lipid contamination.

The results of this study also demonstrate that there are significant variations in the amount of baseplate contamination between component designs. Mason et al reported that different implants have variation in experimental pull-out strength, [16] and Martin et al corroborated these findings. [12] We hypothesize that this observed variation in fluid contamination may be a result of variable keel geometry. We found that one design (Depuy Attune, original baseplate) with a conical keel had higher percent contamination relative to other designs. This design has also been subject to tibial debonding clinically, [[19], [20], [21]] and subsequently underwent design modification by the manufacturer. We also assessed the updated design (S+ baseplate) and did find less contamination relative to its predecessor. It is reassuring, however, that despite variable susceptibility to fluid contamination, it appears that a DB cementation technique effectively eliminates baseplate contamination in all studied designs.

It should be noted that many factors may impact the risk of aseptic loosening and implant debonding in TKA. [22] Cement type, [22] cementation technique, [13,14,22,23] implant design features, [22] as well as some patient factors are all known to influence risk of aseptic loosening. [22] Prior authors have focused on cement mantle thickness as a risk factor for loosening, but in contemporary TKA, the cement-bone interface is rarely the “weak-link”. [11] It is not surprising, then, that an adequate cement mantle is not protective of aseptic loosening at the cement-implant interface. [24] This observation led Cox et al to recommend that efforts should focus on optimization of fixation at the implant-cement interface. [24] To this end, Martin et al found that motion during cement curing can compromise this fixation and they hypothesized that lipid contamination was contributing to observed failures at the cement-implant interface. [13] The present study adds to this existing literature and strongly suggests that cementation technique and component design both influence the degree of fluid contamination of the cement-implant interface.

There are several limitations to this study. Most notably, we utilized an experimental cementation model. This included the use of a white modeling dough rather than bone cement. While this could conceivably impact our observed results, we made this decision intentionally so that the viscosity of the “cement” was uniform across all trials and implants. A trial with polymethylmethacrylate cement demonstrated that, if anything, our use of modeling dough underestimated the difference between cementation techniques. That said, further study will be needed to confirm these results in vivo. Along the same lines, we chose to utilize a fluid volume of 3 drops (0.15 mL) across all trials. It is difficult to know what results we would have obtained if had used more or less fluid. However, we felt this was an appropriate volume as this amount of fluid could very conceivably be introduced at the cement interface during cementation. Additionally, not all tibial components used in this study are still in use in contemporary practice. Despite this, included tibial components were chosen with intention such that variable keel designs were utilized. Finally, while we did attempt to select similar sized implants, given variations in system designs it is possible that small differences in surface area existed between implants. We felt that this slight change would have minimal impact on our results.

## Conclusions

Fluid and lipid contamination of the cement-implant interface varies by cementation technique and implant geometry. Even when 0.15 mL of fluid were present at the keel, greater than 35% of the baseplate was contaminated in multiple designs when a bone-only cement technique was utilized. These results, taken in conjunction with prior data, suggest that both the bone and implant surfaces should be completely coated with cement prior to implant impaction and following impaction the knee should be held with an axial load and no motion. Given the existing variability in cementation technique, [17,18] this study provides important information for surgeons looking to minimize lipid contamination and subsequent aseptic loosening. Future work should focus on in vivo, clinical confirmation of these results.

## Conflicts of interest

Gregory G. Polkowski reports being a paid consultant for and receiving royalties from Enovis. Bohannon J. Mason reports being a board member of AAHKS Publications Committee; being a part of medical/orthopaedic publications of Elsevier/Journal of Arthroplasty; having stocks in Forums Labs; receiving research support from DePuy Synthes; receiving royalties from DePuy Synthes and MedEnvision; and being a paid consultant for DePuy Synthes and Forums Labs. Jacob Wilson reports being a paid consultant for Zimmer-Biomet. Ryan Martin reports being a paid consultant for DePuy. All other authors declare no potential conflicts of interest.

For full disclosure statements refer to https://doi.org/10.1016/j.artd.2024.101574.

## CRediT authorship contribution statement

**Jacob M. Wilson:** Writing – review & editing, Writing – original draft, Supervision, Software, Resources, Project administration, Methodology, Investigation, Formal analysis, Data curation, Conceptualization. **Aleksander P. Mika:** Writing – review & editing, Software, Methodology, Formal analysis, Data curation. **William W. Gilbert:** Writing – review & editing, Methodology, Formal analysis, Data curation. **J. Bohannon Mason:** Writing – review & editing, Supervision, Methodology, Conceptualization. **Gregory G. Polkowski:** Writing – review & editing, Supervision, Resources, Project administration, Methodology. **J. Ryan Martin:** Writing – review & editing, Supervision, Resources, Project administration, Methodology, Investigation, Data curation, Conceptualization.

## Appendix A Supplementary data

Conflict of Interest Statement for Martin

Conflict of Interest Statement for Mason

Conflict of Interest Statement for Polkowski

Conflict of Interest Statement for Gilbert

Conflict of Interest Statement for Mika

Conflict of Interest Statement for Wilson
