# Supplementary material for: Double-Butter: A Cementation Technique That Significantly Reduces Lipid Contamination of the Tibial Baseplate in Total Knee Arthroplasty
Source: Arthroplast Today. 2024 Dec 7;30:101574. doi: 10.1016/j.artd.2024.101574 (PMC11665463; doi:10.1016/j.artd.2024.101574)
Supplement: Conflict of Interest Statement for Polkowski [file mmc3.docx]

# INDIVIDUAL CONFLICT OF INTEREST STATEMENT

***American Association of Hip and Knee Surgeons***

(Adopted from the American Academy of Orthopaedic Surgeons disclosure statement)

The following form **must be filled out completely and submitted by each author (example, 6 authors, 6 forms).**

**All items require a response. If there is no relevant disclosure for a given item, enter "*None*.”**

**Double-Butter: A Cementation Technique that Significantly Reduces Lipid Contamination of the Tibial Baseplate in Total Knee Arthroplasty**

1. Royalties from a company or supplier (The following conflicts were disclosed)

**Enovis**

2. Speakers bureau/paid presentations for a company or supplier (The following conflicts were disclosed)

**None**

3A. Paid employee for a company or supplier (The following conflicts were disclosed)

**None**

3B. Paid consultant for a company or supplier (The following conflicts were disclosed)

**Enovis**

3C. Unpaid consultants for a company or supplier (The following conflicts were disclosed)

**None**

4. Stock or stock options in a company or supplier (The following conflicts were disclosed)

**None**

5. Research support from a company or supplier as a Principal Investigator (The following conflicts were disclosed)

**None**

6. Other financial or material support from a company or supplier (The following conflicts were disclosed)

**None**

7. Royalties, financial or material support from publishers (The following conflicts were disclosed)

**None**

8. Medical/Orthopaedic publications editorial/governing board (The following conflicts were disclosed)

**None**

9. Board member/committee appointments for a society (The following conflicts were disclosed)

**Board Member**

**Each author must sign AND print or type his/her name, date and submit a separate form**

In addition, one BLINDED Conflict of Interest form (no author names used) should be submitted per manuscript with all author disclosures.

Gregory G. Polkowski Gregory Polkowski 12/17/2023

Author Name (Print or Type) Author Signature Date
